# Coplanar Donor-π-Acceptor Dyes Featuring a Furylethynyl Spacer for Dye-Sensitized Solar Cells

**DOI:** 10.3390/ma12050839

**Published:** 2019-03-12

**Authors:** Luis A. Serrano, Kwang-Won Park, Sungwoo Ahn, Alan A. Wiles, Jongin Hong, Graeme Cooke

**Affiliations:** 1 WestCHEM, School of Chemistry, University of Glasgow, Glasgow G12 8QQ, UK; llaassgg5@hotmail.com (L.A.S.); alan.wiles@glasgow.ac.uk (A.A.W.); 2 Department of Chemistry, Chung-Ang University, Seoul 06974, Korea; bryan.kwangwon.park@gmail.com (K.-W.P.); ahn2788@gmail.com (S.A.)

**Keywords:** dye-sensitized solar cell, donor-π-acceptor, furyethynyl, density functional theory

## Abstract

Coplanar metal-free organic dyes featuring a furylethynyl spacer with different donor residues (MeO-, MeS-, and Me_2_N-) have been synthesized. Density functional theory (DFT) calculations predicted that the Me_2_N- residue would facilitate more effective charge transfer from donor to acceptor than the MeO- and MeS- residues. In agreement with DFT calculations, the dye-sensitized solar cells (DSSCs) fabricated with the Me_2_N- functionalized dye exhibited the best power conversion efficiency (η), 2.88%. Furthermore, the effect of the furan spacer on the photophysical properties and DSSC parameters are discussed in comparison to a previously reported thiophene counterpart.

## 1. Introduction

Incoming solar irradiation possesses a huge amount of energy and as a consequence, photovoltaic (PV) devices have been extensively developed to meet our ongoing demand for clean energy. Among next-generation PV devices, dye-sensitized solar cells (DSSCs) have been extensively explored because of their tunable color/transparency and good performance under low light and variable solar incident angles [1,2]. Photosensitizers play a prominent role in harvesting sunlight and mediating the interaction between a wide-bandgap semiconductor and a redox shuttle. Recently, metal-free organic photosensitizers have embraced a donor-π bridge-acceptor (D-π-A) structure, which promotes intramolecular charge separation and effective injection of excited electrons to the conduction band of the semiconductor. They also have notable advantages, such as high molar extinction coefficients, rich synthetic protocols, and cost-effective mass production. In these systems, dialkylamine or diphenylamine moieties are commonly used as the electron donor and a cyanoacrylic acid acts as the electron acceptor and anchoring unit [3,4,5]. However, various π-conjugation segments have been tested to modulate the photophysical properties of the metal-free D-π-A.

Chalcogenophene units, such as furan, thiophene, and selenophene, have been employed as stable and efficient heterocycle units because of effective π-conjugation, low resonance stabilization energy, and a bathochromic shift in light absorption [6,7,8]. For example, Li et al. have indicated that furan as the π-bridge provides a negligible dihedral angle with adjacent molecular units and thus results in a higher open circuit voltage (*V_oc_*) in the DSSCs than thiophene and selenophene [7]. Qu et al. reported that the diketopyrrolopyrrole (DPP) dye containing a furan bridge exhibits better stability and photovoltaic performance than that with benzene or thiophene [8]. Recently, furan has been reported to give rise to greater DSSC efficiency in porphyrin dyes when compared to thiophene [9]. Despite these studies, the furan moiety has not been widely employed, and knowledge of its effect on photophysical and photovoltaic properties still is lacking for coplanar dyes. We propose that furan would play a vital role as a π-spacer in metal-free D-π-A coplanar photosensitizers since it exhibits superior coplanarity compared to other heterocycle units. Accordingly, the electron donor group will play a more significant role than that of nonplanar molecules. We report the synthesis of three metal-free organic compounds featuring a furylethynyl spacer and their optical and electrochemical properties, which have been compared to their DFT-predicted values. We also investigate how different donor groups (MeO-, MeS-, and Me_2_N-) affect the photophysical properties of the synthesized dyes and the photovoltaic performance of the resultant DSSCs. Furthermore, we compare the influence of furan and thiophene on Me_2_N-dye properties and DSSC performance.

## 2. Results and Discussion

### 2.1. Synthesis

**Scheme 1:**
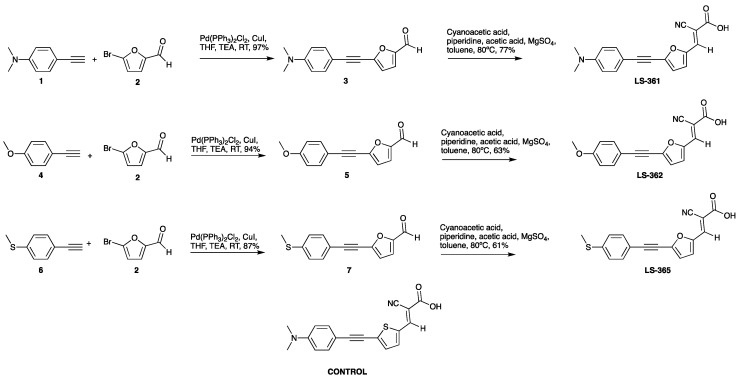
Synthetic route toward dyes **LS-361**, **LS-362**, and **LS-365**.

The synthesis of the dyes used in this study is outlined in Scheme 1. Building blocks **3**, **5**, and **7** were synthesized using Sonogashira reactions, which upon subsequent Knoevenagel condensation with cyanoacrylic acid provided dyes **LS-361**, **LS-362**, and **LS-365** in good overall yields.

### 2.2. Characterization

**Figure 1:**
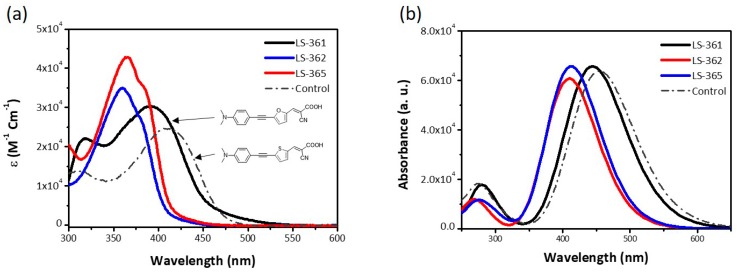
(**a**) UV-vis absorption spectra of Control, **LS-361**, **LS-362**, and **LS-365** dyes in dimethylformamide (DMF) solution (1.5 × 10^−5^ M) and (**b**) their calculated absorption spectra at CAM-B3LYP/6-311++G (d,p) level of the theory under acetonitrile using PCM.

The UV-vis spectra of the three compounds recorded in dimethylformamide (DMF) are provided in Figure 1a. Two absorption peaks were seen in **LS-361**, whereas one absorption peak was observed in **LS-362** and **LS-365**. The absorption band of **LS-361** around 320 nm is likely due to the π-π* transition of the conjugated aromatic moieties, while the absorption bands around 360 nm (for **LS-362** and **LS-365**) and around 392 nm (for **LS-361**) correspond to an intramolecular charge transfer (ICT) between the donor and acceptor groups. Recently, we reported an organic dye featuring a thienylethynyl spacer (denoted as **Control**) analogous to **LS-361** [10,11]. The wavelength of absorption maxima decreases in the order of **Control** > **LS-361** > **LS-365** > **LS-362** in agreement with the electronic absorption spectra from TDDFT calculations shown in Figure 1b. This trend is in line with the better electron donating ability of the dimethylamino moiety, which increases ICT behaviors in D-π-A molecules. The different electronegativity of the heteroatoms between **Control** and **LS-361** influences the absorption peak position and molar extinction coefficient [7]. This change in electronegativity results in the red-shift of the absorbance maximum. However, the extinction coefficient of **LS-361** is higher than that of **Control**. It is likely due to the decrease in the torsion angle between the ethynyl unit and the heterocycle (i.e., 0.66° for furan and 0.78° for thiophene).

**Figure 2:**
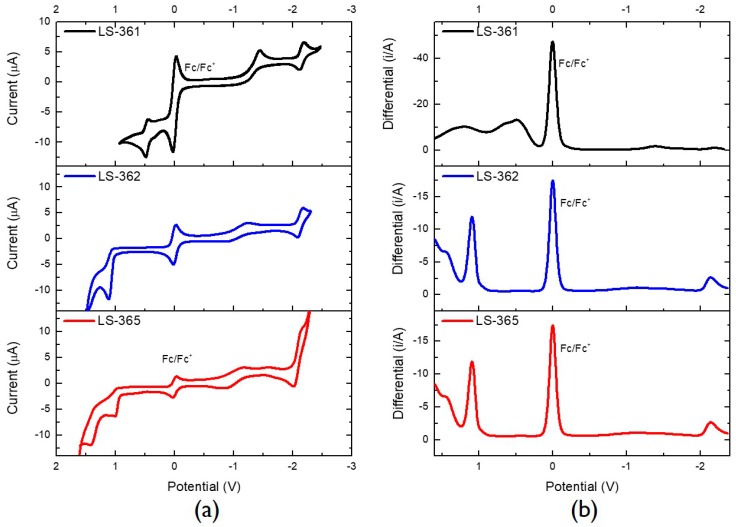
(**a**) Cyclic voltammetry (CV) and (**b**) square wave voltammetry (SWV) plots of **LS-361**, **LS-362*,*** and **LS-365** dyes recorded in DMF (1.0 × 10^−3^ M).

The electrochemical properties of the dyes were explored using cyclic voltammetry (CV) and square wave voltammetry (SWV) (Figure 2), and their estimated ionization potentials (IP) and electron affinities (EA) obtained from the SWV measurements are provided in Table 1 [12]. The stronger donating ability of the amino moiety allowed for more up-shift of the IP energy of **LS-361**, which is consistent with the oxidation potential (***E***_ox_) obtained from CV and SWV, and, thus, the smaller energy gap (*E_gap_*). *E_gap_* increases in the order of **Control** > **LS-361** > **LS-365** > **LS-362**, and this is consistent with the trend seen in the optical bandgap (*E_opt_*) measurements.

### 2.3. Theoretical Calculations

Density functional theory (DFT) and time-dependent DFT (TDDFT) calculations were conducted to gain insight into the electronic structure and optical properties of the organic dyes [13]. Figure S1 illustrates that all of the optimized molecular geometries are entirely coplanar. It originates from the rigid nature of a carbon-carbon triple bond (C≡C) and conjugated (hetero)aromatic moieties (i.e., phenyl and furan groups). Interestingly, all of the dihedral angles between the carbon chains are less than 1°. This coplanar structure is conducive to the stability of the molecule and the intramolecular charge transfer (ICT) between donor and acceptor groups. Second order perturbation theory (SOPT) analysis of the Fock matrix on the natural bond orbital (NBO) basis can determine the charge transfer characteristics between different parts of the molecule [14]. Specific carbon atoms were selected to investigate the electronic delocalization process, and they are numbered (C_1_~C_8_). Table S1 summarizes the NBO parameters, conjugative interaction energies (Δ*E*) between the π and π* orbitals, the energy difference between the interacting NBO and matrix element (*E_acc_-E_don_*), and the off-diagonal element associated with the NBO Fock matrix (*F(acc,don)*). High conjugative interaction energies imply more charge transfer from donor to acceptor parts. Table S2 summarizes the NBO population charge for the electron donor, π-bridge, and electron acceptor, which are denoted as *q^donor^*, *q^π-bridge^*, and *q^acceptor^*, respectively. The most significant charge variance between the natural charges on the donor and acceptor groups is represented as Δ*q^D^^-A^*. In this study, both Δ*E* and Δ*q^D^^-A^* values can be used to determine the electron donating ability of the moiety. The values of **LS-361** are higher than those of **LS-362** and **LS-365,** implying that the Me_2_N- moiety facilitates more effective charge transfer from the donor to the acceptor than the MeO- and MeS- moieties.

**Figure 3:**
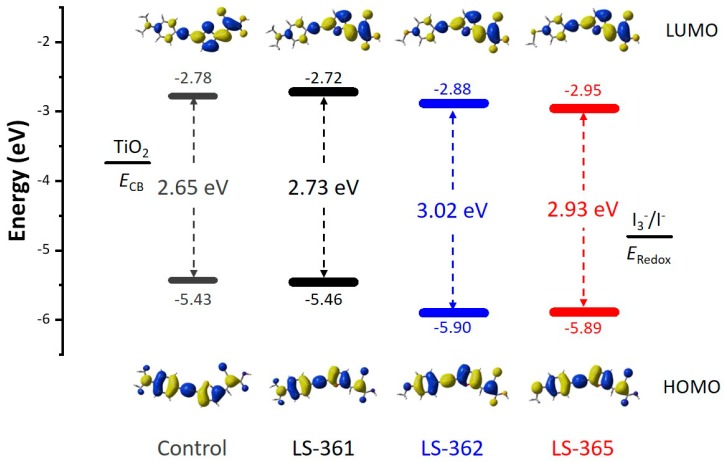
Calculated frontier molecular orbitals and energy levels of **Control**, **LS-361**, **LS-362**, and **LS-365** dyes.

The highest occupied molecular orbital (HOMO) is associated with the electron donating ability of a molecule, and the lowest unoccupied molecular orbital (LUMO) represents its electron accepting ability. Figure 3 displays the frontier molecular orbitals from HOMO to LUMO energy state of the three dyes (**LS-361**, **LS-362,** and **LS-365**) and the **Control**. At the HOMO level, electrons are extended from the donor to the acceptor. On the contrary, at the LUMO level, the excited electron is localized in the π-conjugation and acceptor groups. This spatial molecular orbital distribution is beneficial to the photo-driven ICT process. For DSSC operation, the HOMO of the molecule should be located below the iodide redox potential, and its LUMO should be above the conduction band (CB) of the TiO_2_ semiconductor [1]. Interestingly, the calculated energy levels reveal that **LS-361** is entirely different from **LS-362** and **LS-365**. Table 2 summarizes the absorption wavelengths and oscillator strengths calculated at the CAM-B3LYP level. The excitation at λ_max_ is mainly due to a HOMO→LUMO transition and, thus, it can be interpreted as the ICT peak. The red-shift transitions can be seen as the donor strength increases. Although the oscillator strength (*f*) is sensitive to the basis set choice, a large f indicates large light harvesting efficiency (*LHE*) that is directly related to short-circuit current density (*J_sc_*) [14].

### 2.4. Photovoltaic Performance

**Figure 4:**
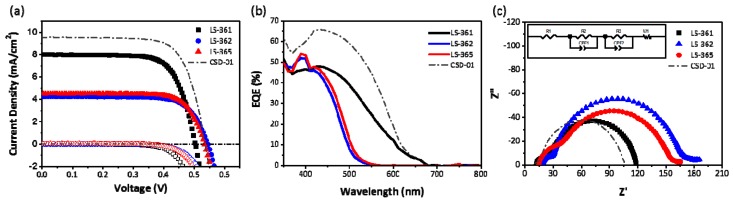
(**a**) J-V characteristics, (**b**) incident photon-to-current efficiency (IPCE) spectra, and (**c**) Nyquist plots in the dark of the dye-sensitized solar cells (DSSCs) sensitized with **LS-361**, **LS-362**, and **LS-365** dyes. Those results of the **Control** (grey dotted line) are provided for comparison. The equivalent circuit model for EIS data fitting is inserted.

Figure 4a shows J-V characteristics of DSSCs fabricated from the three dyes. The photovoltaic parameters are summarized in Table 3. **LS-361** exhibited the best photovoltaic performance, although its open-circuit voltage (*V_oc_*) was lower than that of **LS-362** and **LS-365**. The considerable increase in short-circuit current density (*J_sc_*) coincides with the computational prediction described above. Dark current in the DSSC mainly results from the loss of the injected electron from TiO_2_ to *I*_3_^-^ and the dark current is related to the *V_oc_* under illumination. Contrary to our initial hypothesis, the photovoltaic performance of the **Control** is better than that of **LS-361**, even if the former has worse light harvesting ability than the latter. Figure 4b shows incident photon-to-current efficiency (IPCE) spectra for the DSSCs. The IPCE of **LS-361** displays a broader band between 300 nm and 700 nm than that of **LS-362** and **LS-365**. It is in accordance with the UV-vis spectra in solution mentioned above. It should be noted that there is a significant drop in overall efficiency between **LS-361** and **Control**, likely due to self-quenching of excitons and charge recombination induced by close π-π aggregation between coplanar dye molecules [15].

Figure 4c shows the Nyquist plots of the electrochemical impedance spectroscopy (EIS) analysis in the dark. The EIS parameters estimated by a pertinent equivalent circuit are summarized in Table S3. The first semicircle in the high-frequency region corresponds to the reaction of the iodine/iodide redox couple at the interface between the counter electrode and the electrolyte. The second semicircle in the middle frequency region reflects the charge recombination between ions in the electrolyte and electrons in the TiO_2_ semiconductor [16,17]. The radius of the second semicircle decreases in the order of **LS-362** > **Control** > **LS-365** > **LS-361**, yielding recombination resistances (*R_rec_*) of 126.00 Ω, 92.51 Ω, 85.08 Ω, and 75.91 Ω, respectively. The back reaction between photoinjected electrons was more suppressed in the electrolyte/dye/TiO_2_ interface of **LS-362**. It is well known that the dye itself can suppress the dark current at the electrolyte/TiO_2_ interface by forming a blocking layer [5,11]. Accordingly, it is likely that MeO-, which is comprised of highly electronegative lone pairs, may inhibit recombination toward redox electrolytes on the TiO_2_ surface. This not only corresponds to the trend in the dark current in Figure 4a, but also explains the *V_oc_* of **LS-362** and **LS-365**, 0.544 V and 0.535 V, respectively. Interestingly, **LS-362** exhibited better charge-collection efficiency (*η_cc_*) than **LS-361** and **LS-365**. We think that the MeO- substituent with two lone pair electrons is more useful in preventing the charge recombination mentioned above. Nevertheless, the Me_2_N- substituent leads to the best power conversion efficiency due to considerable light harvesting ability and strong electron donating ability despite fast charge recombination [18].

## 3. Materials and Methods

### 3.1. General

Nuclear magnetic resonance spectra were recorded on a Bruker AVIII (400 MHz) spectrometer (Billerica, MA, USA), and the chemical shifts are relative to tetramethylsilane and are reported in ppm. Mass spectrometry was either recorded on a Thermo Scientific LTQ Orbitrap XL (Waltham, MA, USA) using electrospray (negative mode, ESI, or a JEOL-700 MStation (EI, Akishima, Tokyo, Japan). Infrared spectra were recorded on a Perkin-Elmer FTIR (Waltham, MA, USA) and transmittance maxima are reported in wavenumbers (cm^−1^). UV-vis absorbance spectra were recorded on a JASCO UV/Vis/NIR spectrophotometer (Tokyo, Japan) and the estimated optical bandgaps (*E_g_*) were determined using the absorption edge of the longest wavelength absorption (λ) using equation *E_g_* = 1240/λ. The solution electrochemistry measurements were undertaken using a CH Instruments 440 A electrochemical analyzer (Austin, TX, USA). A platinum working electrode, a platinum wire counter electrode, and a silver wire pseudo-reference electrode were used in all electrochemical measurements. Ferrocene was used as an internal standard. The redox couples are reported versus the ferrocene/ferrocenium (*Fc/Fc^+^* = −4.8 eV) redox couple (adjusted to 0.0 V). The supporting electrolyte used was electrochemical grade tetrabutylammonium hexafluorophosphate (0.1 M) and was dissolved in dry dimethylformamide. Electrochemical data were recorded following purging the solution with nitrogen gas for 3 min. All chemicals and solvents were obtained from suppliers and used without prior purification, Sigma-Aldrich (UK), TCI (Europe) or Alfa-Easar (UK).

### 3.2. Syntheses

To synthesize 5-((4-(dimethylamino)phenyl)ethynylfuran-2-carbaldehyde **(3)**, 5-bromo-2-furaldehyde **2** (300 mg, 1.71 mmol) and 1-ethynyl-4-dimethylaniline **1** (0.32 g, 2.23 mmol) were dissolved in dry triethylamine (15 mL) and dry tetrahydrofuran (15 mL) under N_2_ atmosphere. The solution was degassed with N_2_ for 20 min. Pd(PPh_3_)_2_Cl_2_ (36 mg, 3% mmol) and CuI (9.8 mg, 3% mmol) were then added to the mixture and the solution was stirred for 1 hour at room temperature. The reaction was diluted with dichloromethane (40 mL) and washed with brine (3 × 40 mL). The organic layer was dried over MgSO_4_, filtered, and the solvent removed under reduced pressure. The crude product was purified by column chromatography (SiO_2_, petroleum ether:dichloromethane; 1:1) to yield compound **3** as a yellow-orange solid (387 mg, 97%); M.p. 130–131 °C; ^1^H NMR (500 MHz, CDCl_3_) δ = 2.92 (3H, s), 6.56 (2H, dt, *J* = 8.9 Hz, *J* = 4.3 Hz), 6.59 (1H, d, *J* = 3.7 Hz), 7.16 (1H, d, *J* = 3.7 Hz), 7.33 (2H, dt, *J* = 8.9 Hz, *J* = 4.3 Hz), 9.50 (1H, s); ^13^C NMR (125 MHz, CDCl_3_) δ = 40.0, 77.1, 98.7, 107.3, 111.7, 115.7, 121.8, 133.1, 143.2, 150.9, 152.0, 176.9; *m/z* (ESI^+^) 262.0838 [M + Na^+^] (C_15_H_13_NO_2_Na requires 262.0829).

To synthesize 5-((4-methoxyphenyl)ethynyl)furan-2-carbaldehyde **(5)**, 5-bromo-2-furaldehyde **2** (300 mg, 1.71 mmol) and 1-ethynyl-4-methoxybenzene **4** (0.29 mL, 2.23 mmol) were dissolved in dry triethylamine (10 mL) and dry tetrahydrofuran (10 mL) under an N_2_ atmosphere. The solution was degassed with N_2_ for 20 min. Pd(PPh_3_)_2_Cl_2_ (36.0 mg, 3% mmol) and CuI (9.8 mg, 3% mmol) were then added to the mixture and the solution was stirred overnight at room temperature. The reaction was then diluted with dichloromethane (40 mL) and washed with brine (3 × 40 mL). The organic extract was dried over MgSO_4_, filtered, and the solvent removed under reduced pressure. The crude product was purified by column chromatography (SiO2, petroleum ether:ethyl acetate; 2:1) to yield compound **5** as a pale yellow solid (330 mg, 87%); M.p. 119–120 °C; ^1^H NMR (500 MHz, CDCl_3_) δ = 3.73 (3H, s), 6.63 (1H, d, *J* = 3.6 Hz), 6.80 (2H, dt, *J* = 9.0 Hz, *J* = 4.8 Hz), 7.16 (1H, d, *J* = 3.6 Hz), 7.40 (2H, dt, *J* = 9.0 Hz, *J* = 4.8 Hz), 9.51 (1H, s); ^13^C NMR (125 MHz, CDCl_3_) δ = 55.3, 77.5, 96.8, 113.1, 114.5, 116.4, 121.6, 133.6, 142.4, 153.3, 160.3, 177.0; *m/z* (ESI^+^) 249.0522 [M + Na^+^] (C_14_H_10_O_3_Na requires 249.0515).

To synthesize 5-((4-(methio)phenyl)ethynyl)furan-2-carbaldehyde **(7)**, 5-bromo-2-furaldehyde **2** (360 mg, 2.06 mmol) and 1-ethynyl-4-thioanisole **6** (0.4 g, 2.67 mmol) were dissolved in dry triethylamine (15 mL) and dry tetrahydrofuran (15 mL) under N_2_ atmosphere. The solution was degassed with N_2_ for 20 min. Pd(PPh_3_)_2_Cl_2_ (43 mg, 3% mmol), and CuI (12 mg, 3% mmol) were then added to the mixture and the solution was stirred overnight at room temperature. The reaction was then diluted with dichloromethane (40 mL) and washed with brine (3 × 40 mL). The organic layer was dried over MgSO_4_, filtered, and the solvent removed under reduced pressure. The crude product was purified by column chromatography (SiO_2_, petroleum ether:ethyl acetate; 2:1) to yield compound **5** as a yellow-orange solid (467 mg, 94%); M.p. 125–126 °C; ^1^H NMR (500 MHz, CDCl_3_) δ = 3.43 (3H, s), 6.69 (1H, d, *J* = 3.7 Hz), 7.15 (2H, dt, *J* = 8.5 Hz, *J* = 3.5 Hz), 7.18 (1H, d, *J* = 3.6 Hz), 7.38 (2H, dt, *J* = 8.5 Hz, *J* = 3.5 Hz), 9.56 (1H, s); ^13^C NMR (125 MHz, CDCl_3_) δ = 15.1, 78.6, 96.5, 116.8, 117.1, 121.4, 125.7, 132.0, 132.0, 142.4, 152.4, 177.1; *m/z* (ESI^+^) 265.0294 [M + Na^+^] (C_14_H_10_O_2_SNa requires 265.0281).

To synthesize 2-cyano-3-(5-((dimethylaniline)ethynyl)furan-2-yl)acrylic acid **(LS-361)**, 5-((4-(dimethylamino)phenyl)ethynylfuran-2-carbaldehyde **3** (200 mg, 0.84 mmol), cyanoacetic acid (85 mg, 1.00 mmol), piperidine (8.3 μL, 0.084 mmol), acetic acid (29 μL, 0.5 mmol), and MgSO_4_ (41 mg, 0.17 mmol) were stirred in dry toluene (40 mL) under N_2_ atmosphere and the solution was heated at 80 °C for 2 hours. The reaction was cooled to room temperature. The precipitate was filtered and washed with petroleum ether(3 × 20 mL) and with water (3 × 20 mL). Recrystallization from methanol gave **LS-361** as a red solid (183 mg, 71%); M.p. 232–233 °C; ^1^H NMR (500 MHz, CDCl_3_) δ = 3.04 (3H, s), 6.66 (2H, d, *J* = 8.9 Hz), 6.72 (1H, d, *J* = 3.6 Hz), 7.37 (1H, d, *J* = 3.6 Hz), 7.44 (2H, d, *J* = 8.9 Hz), 7.95 (1H, s); ^13^C NMR (125 MHz, CDCl_3_) δ = 40.1, 78.0, 99.2, 111.7, 107.6, 116.3, 117.1, 117.2, 121.2, 133.1, 137.3, 142.5, 148.6, 150.7, 161.8); *m/z* (ESI^+^) 305.0925 [M + Na^+^] (C_18_H_13_N_2_O_3_ requires 305.0932).

To synthesize 2-cyano-3-(5-((4-methoxyphenyl)ethynyl)furan-2-yl)acrylic acid **(LS-362)**, 5-((4-methoxyphenyl)ethynyl)furan-2-carbaldehyde **5** (330 mg, 0.33 mmol), cyanoacetic acid (140 mg, 1.59 mmol), piperidine (13 μL, 0.13 mmol), acetic acid (61 μL, 1.06 mmol), and MgSO_4_ (65 mg, 0.27 mmol) were stirred in dry toluene (40 mL) under N_2_ and the solution was heated at 80 °C for 2 hours. The reaction mixture was then cooled to room temperature. The precipitate was filtered and washed with petroleum ether (3 × 20 mL) and with water (3 × 20 mL). Recrystallization from methanol gave **LS-362** as an orange solid (237 mg, 61%); M.p. 190–191 °C; ^1^H NMR (500 MHz, d_6_-DMSO) δ = 3.82 (3H, s), 7.04 (2H, d, *J* = 8.8 Hz), 7.18 (1H, d, *J* = 3.8 Hz), 7.53 (1H, d, *J* = 3.8 Hz), 7.58 (2H, d, *J* = 8.8 Hz), 8.02 (1H, s); ^13^C NMR (125 MHz, d_6_-DMSO) δ = 56.0, 78.3, 97.9, 100.0, 112.4, 115.1, 116.3, 119.5, 124.9, 133.9, 137.7, 141.1, 149.2, 161.0, 163.9; *m/z* (ESI^+^) 316.0568 [M + Na^+^] (C_17_H_11_NO_4_Na requires 316.0580).

To synthesize 2-cyano-3-(5-(((4-methylthio)phenyl)ethynyl)furan-2-yl)acrylic acid **(LS-365)**, 5-((4-methylthio)phenyl)ethynylfuran-2-carbaldehyde **7** (330 mg, 0.33 mmol), cyanoacetic acid (140 mg, 1.59 mmol), piperidine (13 μL, 0.13 mmol), acetic acid (61 μL, 1.06 mmol), and MgSO_4_ (65 mg, 0.27 mmol) were stirred in dry toluene (40 mL) under N_2_ atmosphere and the solution was heated at 80 °C for 2 hours. The reaction mixture was then cooled to room temperature. The precipitate was filtered and washed with petroleum ether (3 × 20 mL) and with water (3 × 20 mL). Recrystallization from methanol gave **LS-365** as an orange solid (264 mg, 63%); M.p. 204–205 °C; ^1^H NMR (500 MHz, d_6_-DMSO) δ = 2.53 (3H, s), 7.21 (1H, d, *J* = 3.7 Hz), 7.34 (2H, d, *J* = 8.5 Hz), 7.54 (3H, m), 8.03 (1H, s). ^13^C NMR (125 MHz, d_6_-DMSO) δ = 14.6, 79.3, 93.1, 97.5, 100.4, 116.3, 119.9, 124.7, 132.3, 126.0, 137.7), 140.9, 142.3, 149.4, 163.7; *m/z* (ESI^+^) 308.0382 [M − H^+^] (C_17_H_10_NSO_3_ requires 308.0387).

### 3.3. Computational Details

All of DFT and TDDFT calculations were carried out with the Gaussian ’09 package (Wallingford, Connecticut, USA) [19]. The ground-state geometries were fully optimized at the DFT level of B3LYP functional using 6-311G ++(d, p) basis set on all atoms without additional diffused function. Frequency calculations were performed at the same level of theory as the geometry optimization to prove that the optimized geometry corresponds to the lowest point of the potential energy surface. NBO characteristics were obtained by the SOPT analysis. The excitation energies and oscillator strengths for the lowest 20 singlet-singlet transitions on the basis of the ground-state geometry were calculated with the TDDFT method under CAM-B3LYP functional and 6-311G++(d, p) basis set. Solvent atmosphere parameters were considered only for TDDFT calculations using the PCM model (Acetonitrile: ɛ = 35.688).

### 3.4. DSSC Fabrication and Photovoltaic Measurements

All acetylene-black TiO_2_ paste was prepared using a paste blending method [20]. The paste was screen-printed onto transparent fluorine-doped SnO_2_ (FTO)-coated conducting glass (TEC 8, Pilkington, 2.2-mm-thick, sheet resistance = 8 Ω/sq). The resulting layer was placed in a muffle furnace (Thermo Scientific, Waltham, MA, USA) and gradually heated to 300 °C over a 30 min period, heated at 300 °C for 1 h, heated to 575 °C for 1 h, and then cooled to room temperature after 3 h. After that, another paste composed of 400 nm TiO_2_ nanoparticles was screen-printed as a scattering layer. Then, an approximately 15-μm-thick nanocrystalline TiO_2_ photoanode could be achieved. The active areas of the electrodes were 0.2025 cm^2^. The prepared TiO_2_ electrodes were immersed in a 0.04 M TiCl_4_ aqueous solution at 75 °C for 30 min. After that, they were rinsed several times with deionized water and ethanol followed by heat treatment at 500 °C for 30 min on a hot plate. O_2_ plasma treatment was conducted to the electrodes for 10 min, and then they were immersed in 0.1 M HNO_3_ solution for 30 min to facilitate dye adsorption. The final photoanodes were immersed in one of three dye-containing ethanol solutions (0.5 mM) for 12 h. The Pt counter electrodes were prepared on the FTO-coated glass with spin-casting of 40 mM of chloroplatinic acid (H_2_PtCl_6_) solution in 2-propanol, followed by thermal annealing at 525 °C for 1 h in a muffle furnace, and two holes were drilled in the glass. Both the dye-sensitized TiO_2_ photoanode and Pt counter electrode were sealed with a 25-μm-thick layer of Surlyn (Solaronix, Switzerland). An iodide-based redox electrolyte (Iodolyte AN-50, Solaronix, Aubonne, Switzerland) was injected into the rear side of the counter electrode.

The photovoltaic characteristics of the devices were measured using a solar cell I-V measurement system (K3000 LAB, McScience, Suwon, Korea) under AM1.5 global one sun illumination (100 mW/cm^2^). Photocurrent density (*J*_sc_), open-circuit voltage (*V*_oc_), fill factor (*FF*), and power conversion efficiency (*η*) were measured simultaneously. The incident photon-to-current conversion efficiency (IPCE) was recorded under monochromatic beam using a spectra IPCE measurement system (K3100, McScience, Suwon, Korea). Electrochemical impedance spectroscopy (EIS) experiments were performed using a frequency response analyzer (Solartron 1260, AMETEK, Leicester, UK). Measurements were performed with cells biased to *V*_oc_ under dark. A sinusoidal potential perturbation with an amplitude of 10 mV was applied over a frequency range from 100 kHz to 0.1 Hz. The recorded spectra were fitted using appropriate equivalent circuit models built in the ZView complex nonlinear least-square regression software (AMETEK, Leicester, UK).

## 4. Conclusions

In summary, we report the facile synthesis of coplanar metal-free organic dyes featuring a furylethynyl spacer with different donor residues (MeO-, MeS- and Me_2_N-). The Me_2_N- residue facilitates more effective charge transfer from donor to acceptor than the MeO- and MeS- residues. Likewise, DSSCs with the Me_2_N- functionalized dye exhibited the highest power conversion efficiency of the series (*η* = 2.88%). However, we have found that better planarity, which is beneficial to light absorption properties, can allow for aggregating dyes on the TiO_2_ surface. This may be circumvented in future studies by the addition of additives or co-adsorption with complementary dyes. We hope that this article will encourage the use of the coplanar organic dyes for lower cost photovoltaic applications.

## Figures and Tables

**Table 1:** Summary of optical and electrochemical properties of the three dyes and Control for comparison.

Dyes	*λ_max_* (nm)	*E_0-0_* (eV)	*E_red_* (V)	*E_ox_* (V)	*IP* (eV)	*EA* (eV)	*E_gap_* (eV)
**LS-361**	317, 392	2.27	−2.19	0.49	−5.29	−2.61	2.68
**LS-362**	360	2.73	−2.13	1.12	−5.92	−2.67	3.25
**LS-365**	366	2.66	−2.08	1.04	−5.84	−2.72	3.12
**Control**	312, 408	2.52	−2.08	0.47	−5.27	−2.72	2.55

**Table 2:** Selected excitation energies, electronic transition configurations, and oscillator strengths for **LS-361**, **LS-362**, **LS-365**, and **Control** at B3LYP/6-311++G (d, p) level in acetonitrile.

Dyes	*λ*_max_ (*E*_ex_)	*f*	*LHE*	Transition Assignment
**LS-361**	444.38(2.79)	1.6246	0.976	H − 1→L + 0(12%) H − 0→L + 0(84%) H − 0→L + 1(3%)
**LS-362**	409.71(3.03)	1.5021	0.969	H − 1→L + 0(6%) H − 0→L + 0(90%)
**LS-365**	412.50(3.01)	1.6247	0.976	H − 1→L + 0(12%) H − 0→L + 0(83%)
**Control**	454.59(2.73)	1.5768	0.974	H − 1→L + 0(10%) H − 0→L + 0(85%) H − 0→L + 1(2%)

**Table 3:** Dye-sensitized solar cell (DSSC) performance parameters of **LS-361**, **LS-362, LS-365**, and **Control**.

Dyes	*V_oc_* (V)	*J_sc_* (mA/cm^2^)	*FF*	*η* (%)
**LS-361**	0.505 ± 0.001	7.97 ± 0.04	71.46 ± 0.19	2.88 ± 0.01
**LS-362**	0.544 ± 0.001	4.26 ± 0.01	73.18 ± 0.46	1.70 ± 0.01
**LS-365**	0.535 ± 0.001	4.48 ± 0.02	74.26 ± 0.21	1.78 ± 0.01
**Control**	0.541 ± 0.001	9.72 ± 0.02	71.52 ± 0.17	3.78 ± 0.01

## Supplementary Materials

The following are available online at https://www.mdpi.com/1996-1944/12/5/839/s1, Figure S1: Optimized molecular geometries, Table S1: NBO parameters, Table S2: NBO population charge, Table S3: EIS parameters.
