# Supplementary material for: Coplanar Donor-π-Acceptor Dyes Featuring a Furylethynyl Spacer for Dye-Sensitized Solar Cells
Source: Materials (Basel). 2019 Mar 12;12(5):839. doi: 10.3390/ma12050839 (PMC6427565; doi:10.3390/ma12050839)
Supplement: Supplementary file 1 [file materials-12-00839-s001.pdf]

materials

Supporting Information

Coplanar Donor-π-Acceptor Dyes Featuring a Furylethynyl Spacer for Dye-Sensitized Solar Cells

Luis A. Serrano [1,†], Kwang-Won Park [2,†], Sungwoo Ahn [2], Alan A. Wiles [1], Jongin Hong [2,*] and Graeme Cooke [1,*]

1 WestCHEM, School of Chemistry, University of Glasgow, Glasgow G12 8QQ, United Kingdom; llaassgg5@hotmail.com (L.A.S.); alan.wiles@glasgow.ac.uk (A.A.W.)
2 Department of Chemistry, Chung-Ang University, Seoul 06974, Republic of Korea; bryan.kwangwon.park@gmail.com (K.W.P.); ahn2788@gmail.com (S.A.)
* Correspondence: hongj@cau.ac.kr (J.H.); Graeme.Cooke@glasgow.ac.uk (G.C.); Tel.: +8228205869 (J.H.); +44143305500(G.C.)
† Equal contribution to this work

Received: 14 February 2019; Accepted: 4 March 2019; Published: 12 March 2019

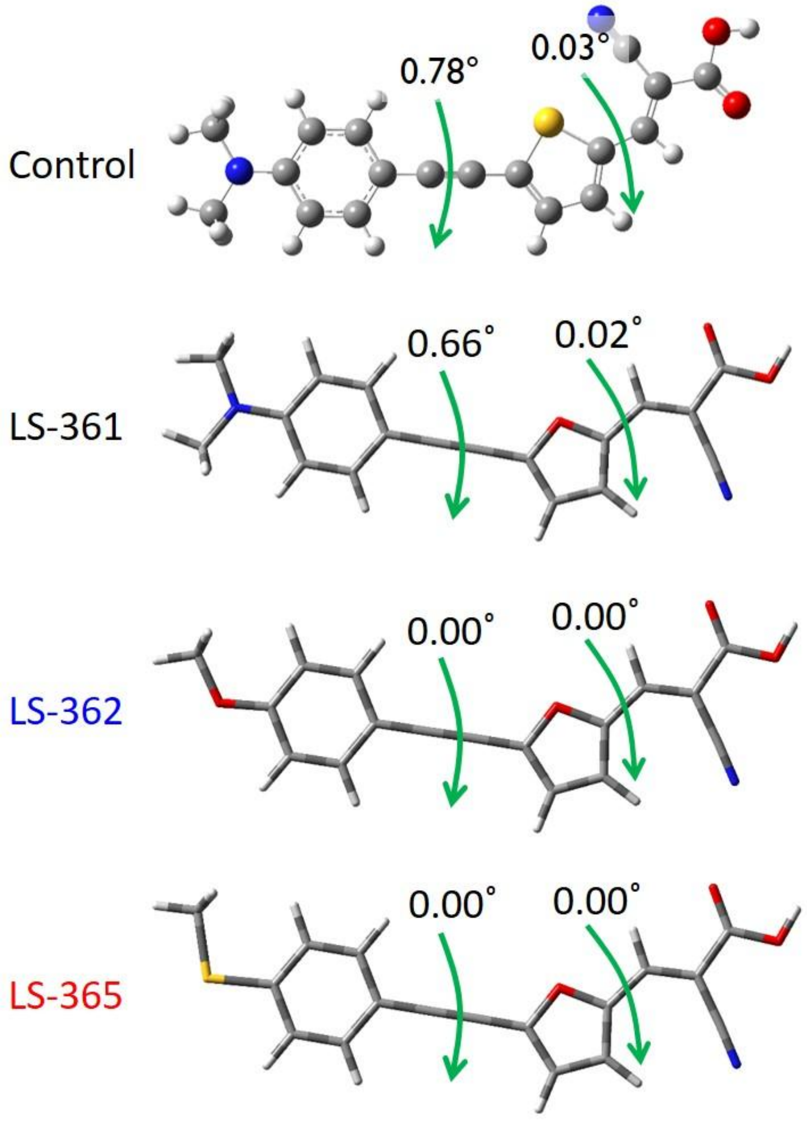

Figure S1. Optimized molecular geometries of the three dyes (LS-361, LS-362 and LS-365).

Table S1. Conjugative interaction energies (*ΔE*) between the π and π* orbitals in LS-361, LS-362 and LS-365 from the second-order perturbation theory analysis of the Fock matrix within the NBO analysis.

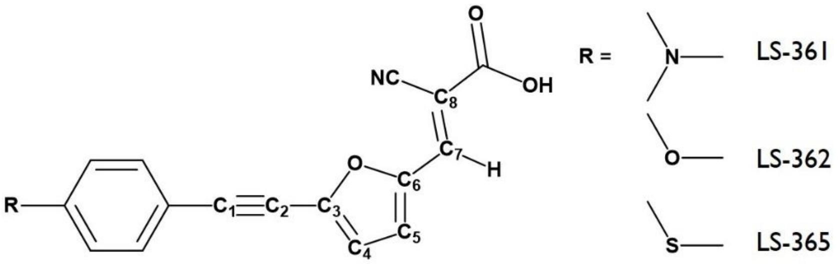

Dye	Donor Orbital	Acceptor Orbital	*ΔE* (kcal/mol)	E_{acc}-E_{don} (a.u.)	*F*(acc,don) (a.u.)
**LS-361**	π(C1≡C2)	π*(C3=C4)	16.86	0.30	0.066
	π(C5=C6)	π*(C7=C8)	23.31	0.29	0.075
**LS-365**	π(C1≡C2)	π*(C3=C4)	15.94	0.30	0.065
	π(C5=C6)	π*(C7=C8)	22.38	0.30	0.073
**LS-362**	π(C1≡C2)	π*(C3=C4)	16.18	0.30	0.065
	π(C5=C6)	π*(C7=C8)	22.63	0.30	0.074
**Control**	π(C1≡C2)	π*(C3=C4)	15.82	0.29	0.064
	π(C5=C6)	π*(C7=C8)	25.26	0.30	0.080

Table S2. The NBO population charge for electron donor, π-bridge and electron acceptor, which denoted as q^{donor}, $q^{\pi\text{-}bridge}$ and $q^{acceptor}$, respectively. $\Delta q^{D\text{-}A}$ represents the charge variance between natural charges on the donor and acceptor groups.

Dye	q^{donor}	$q^{\pi\text{-}bridge}$	$q^{acceptor}$	$\Delta\ q^{D\text{-}A}$
**LS-361**	0.0797	0.1037	-0.1835	0.2632
**LS-362**	0.0492	0.1173	-0.1665	0.2157
**LS-365**	0.0387	0.1223	-0.1610	0.1997
**Control**	0.0836	0.0894	-0.1729	0.2565

Table S3. EIS fitting parameters estimated from the EIS spectra in Figure 4(c).

Dye	R_{tr} (Ω)	R_{rec} (Ω)	C_{μ} (mF)	τ_e (ms)	η_{cc} (%)
**LS-361**	11.26	75.91	0.05	3.56	87.08
**LS-362**	10.09	126.00	0.03	3.56	92.59
**LS-365**	14.75	85.08	0.03	2.52	85.22
**Control**	7.45	92.51	0.04	3.56	93.64
